# *Transcription factor 21* rs12190287 polymorphism is related to stable angina and ST elevation myocardial infarction in a Chinese Population

**DOI:** 10.7150/ijms.89901

**Published:** 2024-01-01

**Authors:** Teng-Hung Yu, Thung-Lip Lee, I-Ting Tsai, Chin-Feng Hsuan, Chao-Ping Wang, Yung-Chuan Lu, Wei-Hua Tang, Ching-Ting Wei, Fu-Mei Chung, Yau-Jiunn Lee, Cheng-Ching Wu

**Affiliations:** 1 Division of Cardiology, Department of Internal Medicine, E-Da Hospital, I-Shou University, Kaohsiung 82445 Taiwan.; 2 School of Medicine, College of Medicine, I-Shou University, Kaohsiung, 82445 Taiwan.; 3 School of Medicine for International Students, College of Medicine, I-Shou University, Kaohsiung, 82445 Taiwan.; 4 Department of Emergency, E-Da Hospital, I-Shou University, Kaohsiung 82445 Taiwan.; 5 Division of Cardiology, Department of Internal Medicine, E-Da Dachang Hospital, I-Shou University, Kaohsiung 807066, Taiwan.; 6 Division of Endocrinology and Metabolism, Department of Internal Medicine, E-Da Hospital, I-Shou University, Kaohsiung 82445 Taiwan.; 7 Division of Cardiology, Department of Internal Medicine, Taipei Veterans General Hospital, Yuli Branch, Hualien 98142 Taiwan.; 8 Faculty of Medicine, School of Medicine, National Yang Ming Chiao Tung University, Taipei 112304 Taiwan.; 9 Division of General Surgery, Department of Surgery, E-Da Hospital, I-Shou University, Kaohsiung 82445 Taiwan.; 10 The School of Chinese Medicine for Post Baccalaureate, College of Medicine, I-Shou University, Kaohsiung 82445 Taiwan.; 11 Lee's Endocrinologic Clinic, Pingtung 90000 Taiwan.; 12 Division of Cardiology, Department of Internal Medicine, E-Da Cancer Hospital, I-Shou University, Kaohsiung 82445 Taiwan.

**Keywords:** transcription factor 21, polymorphism, stable angina, ST elevation myocardial infarction

## Abstract

**Background:** Transcription factor 21 (TCF21, epicardin, capsuling, pod-1) is expressed in the epicardium and is involved in the regulation of cell fate and differentiation via epithelial-mesenchymal transformation during development of the heart. In addition, TCF21 can suppress the differentiation of epicardial cells into vascular smooth muscle cells and promote cardiac fibroblast development. This study aimed to explore whether *TCF21* gene (12190287G/C) variants affect coronary artery disease risk.

**Methods:** We enrolled 381 patients who had stable angina, 138 with ST elevation myocardial infarction (STEMI), and 276 healthy subjects. Genotyping of rs12190287 of the *TCF21* gene was performed.

**Results:** Higher frequencies of the CC genotype were found in the patients with stable angina/STEMI than in the healthy controls. After adjusting for diabetes mellitus, hypertension, age, sex, smoking, body mass index and hyperlipidemia, the patients with the CC genotype of the *TCF21* gene were associated with 2.49- and 9.19-fold increased risks of stable angina and STEMI, respectively, compared to the patients with the GG genotype. Furthermore,* TCF21* CC genotypes showed positive correlations with both stable angina and STEMI, whereas *TCF21* GG genotypes exhibited a negative correlation with STEMI. Moreover, the stable angina and STEMI patients with the CC genotype had significantly elevated high-sensitivity C-reactive protein levels than those with the GG genotype. In addition, significant associations were found between type 2 diabetes mellitus, hypertension, and hyperlipidemia with *TCF21* gene polymorphisms (p for trend < 0.05).

**Conclusion:** *TCF21* gene polymorphisms may increase susceptibility to stable angina and STEMI.

## Introduction

Coronary artery disease (CAD) is still a leading cause of death in industrialized countries even though advances in its prevention and treatment have been made. In the United States, over 18.2 million people have CAD 1,2; in Europe, CAD results in over 3.9 million deaths 3. In Taiwan, more than 17,000 people die of CAD each year 4. Many risk factors contribute to the pathogenesis of CAD, including both modifiable (physical inactivity, diabetes, smoking, hypertension, stress, hypercholesterolemia, and obesity) and non-modifiable (gender, age, race, family history of CAD, and genotype) risk factors 5-10. Behavioral as well as metabolic and environmental risks are leading cause of CAD development 11. Many studies have demonstrated the role of genetic polymorphisms in CAD development, suggesting that a combination of genetic and environmental factors may increase susceptibility to CAD 10,12,13.

Previous genome-wide association studies have reported associations between common variants with the risks of myocardial infarction and CAD 14. Transcription factor 21 (TCF21), a basic helix-loop-helix transcription factor family member, has been demonstrated to play key roles in cell fate and differentiation 15. TCF21 lineage- traced cells were shown to transform into periostin-expressing myofibroblasts in a mice model of myocardial infarction, a process essential to promote adaptive fibrosis and facilitate cardiac healing 16. On the other hand, a lack of cardiac fibroblast production and cardiac repair have been demonstrated in TCF21-deficient mice 17. Furthermore, in a cardiac fibroblast zebrafish model, TCF21 was shown to both contribute to a fibrotic response after injury and also cardiomyocyte proliferation during regeneration 18. In humans of various ethnicity, a higher risk of CAD has been associated with *rs12190287*, a single nucleotide polymorphism (SNP) located in the 3′untranslated region of *TCF21* 19-21. Hamed et al. indicated that the CC genotype and C allele of *TCF21* (12190287G/C) polymorphisms may be genetic risk factors for CAD 22. Furthermore, in a Chinese population, the *TCF21 rs12190287* polymorphism was also shown to confer susceptibility to ventricular septal defects 23. Moreover, a Japanese study reported that *TCF21* rs12190287 (G→C) may be a susceptibility locus for hypertension 24. Another study found significant associations between the GG genotype and G allele of rs12190287 in *TCF21* and an increased TCF21 concentration with the onset and recurrence of paroxysmal atrial fibrillation post ablation 25. Considering these findings, study aimed to further explore whether *TCF21* genetic polymorphisms affected susceptibility to stable angina and ST elevation myocardial infarction (STEMI) and their relationships with clinical and biochemical characteristics.

## Methods

### Participants

Patients with stable angina (n=381) and STEMI (n = 138) were enrolled from the Emergency Room and Cardiovascular Ward at Kaohsiung E-Da Hospital, Taiwan, from January to December 2022. STEMI was identified according to prolonged chest pain (more than 30 minutes), typical increases in circulating biochemical markers (troponin-I and creatine kinase-MB (CK-MB)/creatine-phospho-kinase) with symptoms of ischemia persisting for ≥ 30 min, and ST-segment elevation ≥ 2.0 mm in two or more contiguous electrocardiogram leads. Stable angina was identified as patients with rest or effort-related chest pain which had not progressed in the last 6 months. All of the patients with STEMI underwent primary percutaneous coronary interventions within 12 hours of symptom onset. Patients with renal dysfunction, hepatic dysfunction, heart failure and valvular heart disease were excluded. Healthy controls (n = 276) were unrelated patients who entered the health examination program of the hospital. None the controls had detectable cardiovascular risk factors or were not taking any medication. The Human Research Ethics Committee of E-Da Hospital gave approval for the study, and all of the enrolled patients signed informed consent forms.

### Data collection

All of the enrolled participants lived in southern Taiwan and were of Han Chinese origin. Data on smoking, drinking, and personal disease history were obtained using standardized questionnaires. Smoking and alcohol drinking status were classified as never, former (stopped for at least 1 year), or current. We analyzed former and current drinkers as one group 26. Body mass index (BMI) and waist (between the lowest rib and top of the hip) and hip (at the widest part) circumferences were measured in all of the patients, and the average of two measurements was used to calculate the waist-to-hip ratio. A digital sphygmomanometer (HEM-907, Omron, Japan) was used to measure blood pressure (BP) after a 5-min rest. The modified Simpson's method was used to compute left ventricular ejection fraction with apical 4-chamber views 27. The ATP III criteria were used to diagnose hyperlipidemia as the presence of one of the following: (1) total cholesterol ≥ 200 mg/dL, (2) low-density lipoprotein-cholesterol (LDL-C) ≥ 130 mg/dL, (3) triglyceride level ≥ 150 mg/dL, (4) high-density lipoprotein-cholesterol (HDL-C) < 35/39 mg/dL (men/women), and (5) prescriptions for lipid disorder medications 28. Patients were defined as being hypertensive if they had prescriptions for anti-hypertension medications, systolic BP (SBP) ≥ 140 mmHg, or diastolic BP (DBP) ≥ 90 mmHg. Patients were defined as having diabetes mellitus if they had prescriptions for anti-diabetes medications or a fasting glucose level > 126 mg/dl 29.

### Laboratory measurements

Levels of total cholesterol, LDL-C, complete blood cell count, serum triglycerides, HDL-C, albumin, blood urine nitrogen (BUN), uric acid, and glucose were measured from fasting blood samples 30. The Jaffe method was used to calculate serum creatinine. Hemoglobin A1c was measured using an automated analyzer (HLC-723G8, Tosoh Corp., Tokyo, Japan). CK-MB and troponin I serum levels were measured using chemiluminescent microparticle immunoassays. Total leukocyte count and the lymphocyte, neutrophil and monocytes proportions were measured using an automated cell counter (XE-2100 Hematology Alpha Transportation System; Sysmex, Kobe, Japan). Absolute leukocyte subtype counts were calculated as the product of its proportion and total leukocyte count. The CKD-EPI formula was utilized to determine estimated glomerular filtration rate (eGFR) 31. An immunochemistry system (Beckman Coulter IMMAGE) was used to evaluate plasma levels of high-sensitivity C-reactive protein (hs-CRP). The detection limit was 0.2 mg/L, and all measurements were made twice.

### Determining the *TCF21* genotype (rs12190287)

A QIAamp DNA Blood Mini kit (Qiagen, Valencia, CA) was used to extract genomic DNA from whole blood samples following the manufacturer's protocol. DNA was reconstituted in TE buffer [1 mM EDTA, 10 mM Tris (pH 7.8)], quantified using OD_260_ measurements, and stored at -20°C until use as a PCR template. Genotyping of rs12190287 of the *TCF21* gene was performed by TagMan^®^ SNP Genotyping Assay (Thermo Fisher Scientific) using an Applied Biosystems PRISM7900 Sequence Detection System. In brief, 5 ng of genomic DNA was amplified in a reaction volume of 10 μl containing 0.25 ml of 40× Predesigned TaqMan SNP Genotyping assay (FAM^™^ dye labeled MGB probe + VIC^®^ dye-labeled MGB probe + 2 unlabeled primers), 5 ml of 2× TaqMan^®^ GTXpress™ Master Mix. The cycling conditions were: initial denaturation for 2 minutes at 95°C; 40 cycles at 95°C for 15 seconds, and 60°C for 40 seconds.

### Statistical analysis

Data normality was assessed using the Kolmogorov-Smirnov test. Normally distributed continuous data are shown as means (SD). Non-normally distributed data are shown as median (interquartile range). Plasma triglyceride, BUN, CK-MB, troponin I, and hs-CRP values were logarithmically transformed before the analysis due to skewed distribution. The unpaired Student's *t*-test and *chi*-square test were used to compare means and proportions between the control, STEMI, and stable angina groups when appropriate. A Pearson correlation heat map was employed to investigate the relationship between *TCF21* genotypes and the occurrence of stable angina and STEMI among patients. Associations of genotype frequencies with the risk of stable angina and STEMI were assessed using logistic regression analysis after controlling for age, sex, smoking, BMI, hypertension, hyperlipidemia, and diabetes mellitus, and reported as odds ratios (ORs) with 95% confidence intervals (CIs). Continuous variables among the three genotypes of *TCF21* were compared with one-way analysis of variance. Prevalence rates of type 2 diabetes mellitus (T2DM), hypertension, and hyperlipidemia status and trends in the three genotypes of *TCF21* groups were analyzed using the *chi*-square test and Cochran-Armitage trend test respectably. JMP version 7.0 for Windows (SAS Institute, Cary, NC) was used for the statistical analysis. A p value <0.05 was considered to be statistically significant.

## Results

### Demographic characteristics of the control, stable angina and STEMI groups

The patients with STEMI were significantly older, predominantly male, and had higher rates of hypertension, T2DM, hyperlipidemia, current smoker, drinking, BMI, SBP than the control group. In addition, the patients with stable angina were significantly older, predominantly male, and had higher rates of hypertension, T2DM, hyperlipidemia, current smoker, drinking, BMI, and SBP than the control group. Furthermore, the STEMI group had significantly higher rates of single-vessel disease, stenosis in right coronary and left anterior descending arteries, and higher Gensini score than the stable angina group (Table 1).

### Biochemical characteristics in controls and stable angina or STEMI patients

Patients with STEMI had a significantly higher levels of fasting sugar, HbA1C, total cholesterol, triglyceride, LDL-C, uric acid, BUN, creatinine, total WBC/neutrophil/ monocyte/lymphocyte counts, and hs-CRP, and lower HDL-C, eGFR, and albumin compared to the control group. In addition, the stable angina group had significantly higher fasting sugar, HbA1C, total cholesterol, triglyceride, LDL-C, uric acid, BUN, creatinine, total WBC/neutrophil/monocyte/lymphocyte counts, and hs-CRP, and lower HDL-C, eGFR, and hemoglobin than the control group. Furthermore, the STEMI group had significantly higher fasting sugar, hemoglobin, total WBC/neutrophil/monocyte/lymphocyte counts, CK-MB, and troponin I, and lower HDL-C, eGFR, and albumin than the stable angina group (Table 2).

### Association between *TCF21* genotypes and stable angina and STEMI

Distributions of the *TCF21* gene G/C genotype in the stable angina, STEMI and control groups are shown in Table 3, which shows that they were in Hardy-Weinberg equilibrium. The *TCF21* genotype distributions were significantly different between the stable angina and STEMI groups when compared to the control group (p < 0.01). Higher CC genotype distributions were found in the stable angina and STEMI groups, suggesting an association between *TCF21* gene polymorphisms with stable angina and STEMI (CC vs. GG: crude OR = 2.00, 95% CI = 1.26-3.21, p = 0.004 for stable angina; OR = 4.25, 95% CI = 2.14-9.00, p < 0.0001 for STEMI). These associations between *TCF21* CC genotypes and stable angina/STEMI persisted after logistic regression adjustments for age, sex, smoking, BMI, hypertension, hyperlipidemia, and diabetes mellitus (CC vs. GG: adjusted OR = 2.49, 95% CI = 1.24-5.07, p = 0.010 for stable angina; adjusted OR = 9.19, 95% CI = 4.36-16.33, p < 0.0001 for STEMI).

**Figure 1:**
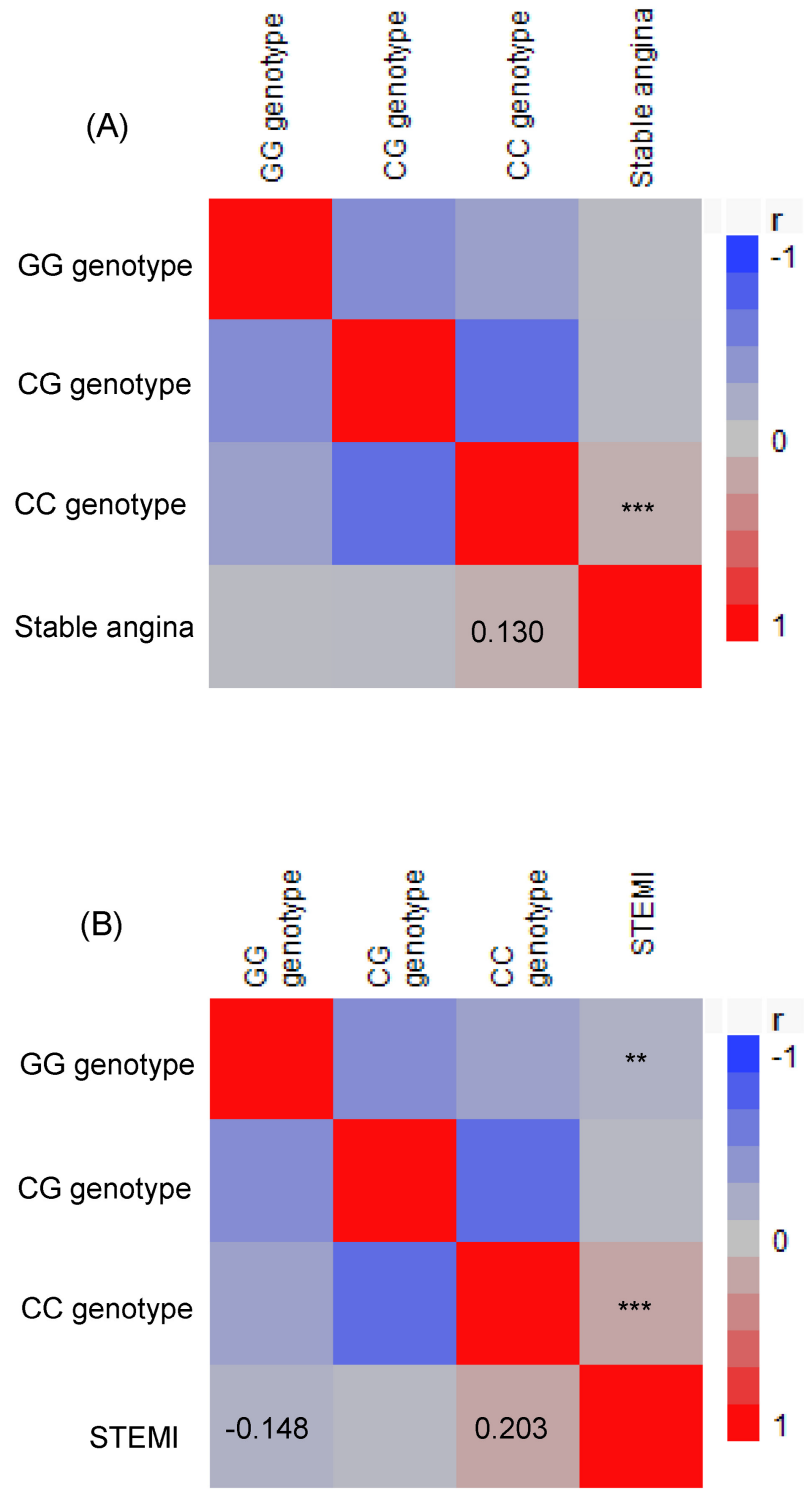
A Pearson correlation heat map was generated to explore the relationship between the genotypes of the *transcription factor 21* and the occurrence of stable angina (A) and ST elevation myocardial infarction (B) among patients. The red color indicates a positive correlation, whereas the blue and grey colors indicate a negative correlation. **p < 0.01 and ***p< 0.001. STEMI, ST elevation myocardial infarction.

Comparing the risk of stable angina and STEMI between the CC vs. GG/GC genotypes showed that the CC genotype was significantly associated with a higher risk (Table 3). Furthermore, using Pearson's correlation analysis, *TCF21* CC genotypes showed positive correlations with stable angina (r = 0.130, p = 0.001) and STEMI (r = 0.203, p < 0.0001), while *TCF21* GG genotypes exhibited a negative correlation with STEMI (r = -0.148, p = 0.003; Figure 1).

### Clinical and biochemical characteristics of the CAD patients by different genotypes of *TCF21*

Table 4 shows the clinical and biochemical characteristics of the 519 enrolled CAD patients according to *TCF21* genotypes. The prevalence rates of GG genotype, GC genotype, and CC genotype were 13.5%, 48.0%, and 38.5%, respectively. The CC genotype group had higher level of hs-CRP than those with GG genotype. No significant differences were found in age, sex, BMI, waist circumference, waist-to-hip ratio, SBP, DBP, fasting glucose, HbA1c, total cholesterol, triglyceride, LDL-C, HDL-C, uric acid, BUN, creatinine, eGFR, hemoglobin, albumin, total WBC count, neutrophil count, monocyte count, and lymphocyte count among the three *TCF21* genotypes.

### Association between *TCF21* genotypes and T2DM, hypertension, and hyperlipidemia status

**Figure 2:**
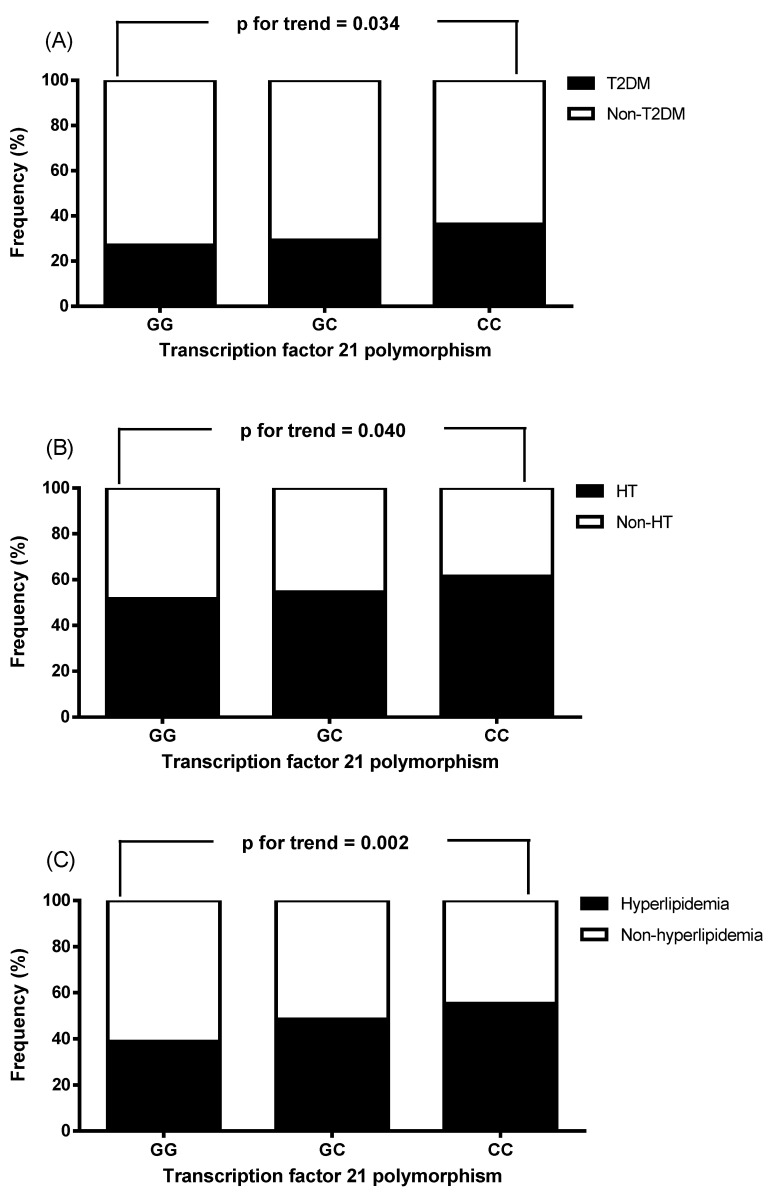
The frequencies of with or without type 2 diabetes mellitus (T2DM), hypertension, and hyperlipidemia status of the all study participates stratified by genotypes of *transcription factor 21* (*TCF21*). T2DM, hypertension, and hyperlipidemia status were significantly associated with genotypes of *TCF21*.

We then analyzed the frequencies of without and with T2DM, hypertension, and hyperlipidemia status of the all study participates stratified by genotypes of *TCF21*, and found that T2DM, hypertension, and hyperlipidemia status were significantly associated with genotypes of *TCF21* (without T2DM vs. with T2DM, GG/GC/CC = 73.0%/70.7%/ 63.7% vs. 27.0%/29.4%/36.3%, p for trend = 0.034; without hypertension vs. with hypertension, GG/GC/CC = 48.4%/45.5%/38.6% vs. 51.6%/54.5%/61.4%, p for trend = 0.040; without hyperlipidemia vs. with hyperlipidemia, GG/GC/CC = 61.1%/51.5%/ 44.6% vs. 38.9%/48.5%/55.4%, p for trend = 0.002, Figure 2).

## Discussion

Our results showed significantly higher distributions of the *TCF21* CC homozygote genotype in the stable angina and STEMI patients compared to the controls. In addition, independent associations between the *TCF21* CC homozygote genotype with stable angina and STEMI were shown in univariate and multivariate logistic regression analyses after adjusting for age, sex, smoking, BMI, hypertension, hyperlipidemia, and diabetes mellitus. Furthermore, *TCF21* CC genotypes demonstrated positive correlations with both stable angina and STEMI, while *TCF21* GG genotypes exhibited a negative correlation with STEMI. Even though previous epidemiological studies have indicated that environmental factors play key roles in the development of cardiovascular disease 32,33, not all people develop CAD 13,34. Molecular epidemiological research has shown that both genetic and environmental factors can influence susceptibility to CAD 35,36.

A previous study reported a significant association between the *TCF21* gene G/C polymorphism with CAD in an Egypt population 22. Santos et al. reported that the* TCF21* polymorphism was a risk factor for CAD events in a Portuguese population 37. Our results further suggest an association between the *TCF21* CC homozygote genotype in both the occurrence of stable angina and STEMI in a Han Taiwanese cohort. The precise role of *TCF21* polymorphisms in stable angina and STEMI has yet to be confirmed, although chronic inflammatory cytokines have been implicated 38-40. Previous studies have shown that chemokines, including macrophage inflammatory protein-alpha, IFN-γ-inducible protein, monocyte chemoattractant protein-1, and eotaxin, play key roles in the pathogenesis of CAD via chronic inflammation 41.

A variation of TCF21 at 6q23.2 has been associated with CAD in Han Chinese and Caucasian populations 35,42. A previous study showed that TCF21 modulates and interacts with aryl-hydrocarbon receptor (AHR), a well-known environmental sensor, and that this regulates the expressions of pro-inflammatory genes in coronary artery smooth muscle cells 43. In addition, the study found that oxidized LDL, a widely recognized contributor to atherosclerosis within plaques, can induce the AHR pathway 43. Taken together with our findings, *TCF21* gene polymorphisms may play important roles with a heritable form of gene-environment interaction in stable angina and STEMI. In addition, Kim et al. also suggested that TCF21 can cooperate with AHR to activate an inflammatory gene expression program that is exacerbated by environmental stimuli, and may contribute to the overall risk of CAD 43. In the present study, we found that the *TCF21* CC genotype group had higher level of hs-CRP than those with *TCF21* GG genotype group. Thus, patients with the *TCF21* gene CC genotype may, through the increased effect of AHR, on inflammatory gene expression activation, contribute to the increased risk of stable angina and STEMI.

The TCF21 gene has been associated with CAD, promoting the stability of plaques and reducing clinical events by modulating the phenotypic transition from smooth muscle cells to fibromyocytes in atherosclerosis 44. The present study shows that *TCF21* gene G/C polymorphism is significantly associated with stable angina and STEMI both in non-adjusted and adjusted models (Table 3). In agreement with our results, Wang et al. 45, Hamed et al. 22 and Schunkert et al. 35 reported significant associations between *TCF21* rs12190287 with CAD. Furthermore, Santos et al. also demonstrated that *TCF21* rs12190287 may be a risk factor for major adverse cardiovascular events, and suggested that the *TCF21* gene may affect fundamental smooth muscle cell processes in response to vascular stress, thereby facilitating the progression of atherosclerosis 37. The mechanisms between rs12190287 risk C allele and CAD could be explained two key pathways. First, TCF21 risk alleles (rs12524865-C or rs12190287-C) may increase the expression of TCF21 on activation of platelet-derived growth factor (PDGF) signaling in coronary vascular smooth muscle cells (VSMCs) 46. PDGF signaling mediated by PDGF receptor beta has been shown to play a major role in epithelial-mesenchymal transition, epicardial fate, VSMC migration, proliferation, and atherosclerosis 47. This increase in PDGF signaling may then promote aggressive formation of plaques in the thoracic aorta and coronary arteries 48. Second, the major risk C variants of *TCF21* rs12190287 preferentially combine with miRNA-224 due to the altered secondary RNA structure, leading to downregulation of the expression of TCF21 49. Furthermore, TGF-β1 and PDGF signaling may be upstream miRNA-224 mediators and influence allele-specific *TCF21* rs12190287 expression. More importantly, the rs12190287 C allele may interact with miR-224 leading to a reduction in TCF21 expression through mechanisms linked to miRNA, playing important roles in the risk of stable angina and STEMI.

In the present study, we showed that T2DM, hypertension, and hyperlipidemia status were significantly associated with genotypes of *TCF21* are consistent with previous studies 22,24. Fujimaki et al. showed that after adjusting for age, sex, BMI and smoking status in multivariable logistic regression analysis, *TCF21* rs12190287 was significantly correlated with hypertension (OR 1.21, p=0.0014), and that the C allele was a risk factor for hypertension 24. Furthermore, Hamed et al. reported that patients with CC genotype predominance showed higher levels of triglycerides, LDL-C, and total cholesterol 22. T2DM, hypertension, and hyperlipidemia were risk factor of atherosclerosis, which contributes to progression of myocardial infarction. Therefore, it is possible that *TCF21* CC genotype may be associated with hypertension, hyperlipidemia, and diabetes mellitus, thereby contributing to risk of stable angina and STEMI in the present study. However, further studies are required to ascertain the role of *TCF21* polymorphism in diabetes mellitus.

Some limitations of this study need to be considered. The sample size was relatively small. In addition, the cross-sectional design limits our ability to infer a causal relationship between *TCF21* gene (12190287G/C) variants and stable angina and STEMI. Long-term follow-up studies are needed to verify the roles of *TCF21* gene polymorphisms with regards to the risk of stable angina and STEMI. Furthermore, as the results of the present study were not replicated, validation of the findings is required in other ethnic groups or in other independent subject panels. Further prospective studies are required to verify our results.

## Conclusions

The results of the current study indicate that the CC genotype of* TCF21* (12190287G/C) polymorphism is associated with stable angina and STEMI. Our findings suggest that *TCF21* rs12190287 polymorphisms could be potential genetic markers for the susceptibility to stable angina and STEMI in Chinese populations.

## Figures and Tables

**Table 1:** Distributions of demographic characteristics in 276 controls and 519 stable angina or STEMI patients

Parameters	Controls	STEMI	Stable angina	p-value^a^	p-value^b^	p-value^c^
N	276	138	381			
Age (years)	58.4 ± 11.2	65.9 ± 12.9	63.9 ± 10.9	< 0.0001	< 0.0001	0.079
Sex (male/female)	72/204	114/24	297/84	< 0.0001	< 0.0001	0.248
Hypertension (n, %)	94 (34.1)	84 (60.9)	265 (69.6)	< 0.0001	< 0.0001	0.063
Type 2 diabetes mellitus (n, %)	49 (17.8)	52 (37.7)	148 (38.9)	< 0.0001	< 0.0001	0.810
Hyperlipidemia (n, %)	42 (15.2)	101 (73.2)	249 (65.4)	< 0.0001	< 0.0001	0.092
Current smoker (n, %)	62 (22.5)	56 (40.6)	157 (41.2)	0.0001	< 0.0001	0.872
Drinking (n, %)	50 (18.1)	39 (28.3)	124 (32.6)	0.016	< 0.0001	0.308
Body mass index (kg/m^2^)	23.7 ± 4.9	25.8 ± 3.7	26.3 ± 3.7	< 0.0001	< 0.0001	0.176
Systolic blood pressure (mmHg)	122 ± 15	134 ± 23	132 ± 20	< 0.0001	< 0.0001	0.247
Diastolic blood pressure (mmHg)	75 ± 12	76 ± 16	77 ± 13	0.534	0.169	0.728
LVMI	-	134.7 ± 49.3	123.4 ± 40.5	-	-	0.059
LVEF (%)	-	59.5 ± 13.4	60.1 ± 13.3	-	-	0.736
Number of diseased vessels (%)						
Single-vessel disease	-	48 (34.8)	98 (25.7)	-	-	0.044
Two-vessel disease	-	45 (32.6)	101 (26.5)	-	-	0.178
Three-vessel disease	-	41 (29.7)	110 (28.9)	-	-	0.866
Location of stenosis				-	-	
Left anterior descending	-	106 (76.8)	251 (65.9)	-	-	0.018
Left circumflex	-	64 (46.4)	171 (44.9)	-	-	0.781
Right coronary artery	-	86 (62.3)	189 (49.6)	-	-	0.011
Gensini score	-	40.0 (14.5-76.5)	22.5 (8.0-52.0)	-	-	0.012

Data are expressed as mean ± SD, number (percentage), or median (interquartile range). STEMI, ST elevation myocardial infarction; LVMI, left ventricular mass index; LVEF, left ventricular ejection fraction; ^a^: Data were compared between ST elevation myocardial infarction patients and controls. ^b^: Data were compared between stable angina patients and controls. ^c^: Data were compared between ST elevation myocardial infarction patients and stable angina patients.

**Table 2:** Biochemical characteristics in 276 controls and 519 stable angina or ST elevation myocardial infarction patients

Parameters	Controls	STEMI	Stable angina	p-value^a^	p-value^b^	p-value^c^
No.	276	138	381			
Fasting sugar (mg/dL)	96.1 ± 31.5	168.3 ± 80.1	136.6 ± 69.9	< 0.0001	< 0.0001	<0.0001
HbA1C (%)	5.7 ± 0.7	7.0 ± 1.9	6.9 ± 1.7	< 0.0001	< 0.0001	0.789
Total cholesterol (mg/dL)	157.2 ± 32.3	181.5 ± 42.6	176.3 ± 45.3	< 0.0001	< 0.0001	0.241
Triglyceride (mg/dL)	84.0 (63.0 - 116.5)	127.0 (86.8 - 197.5)	118.0 (90.0 - 179.0)	< 0.0001	< 0.0001	0.329
HDL-cholesterol (mg/dL)	54.7 ± 15.3	37.0 ± 12.0	40.8 ± 11.0	< 0.0001	< 0.0001	0.001
LDL-cholesterol (mg/dL)	90.9 ± 27.1	110.8 ± 36.0	104.0 ± 38.4	< 0.0001	< 0.0001	0.070
Uric acid (mg/dL)	4.7 ± 1.9	6.8 ± 2.0	6.5 ± 2.2	< 0.0001	< 0.0001	0.348
BUN (mg/dl)	12.0 (9.0 - 15.0)	18.1 (15.6 - 23.7)	17.2 (13.5 - 22.1)	< 0.0001	< 0.0001	0.092
Creatinine (mg/dl)	0.8 ± 0.5	1.5 ± 0.9	1.4 ± 1.1	< 0.0001	< 0.0001	0.534
eGFR (ml/min/1.73m^2^)	124.2 ± 44.3	60.7 ± 24.2	66.4 ± 25.9	< 0.0001	< 0.0001	0.024
Hemoglobin (g/dl)	14.3 ± 1.1	14.0 ± 2.4	13.6 ± 2.0	0.824	0.041	0.046
Albumin (g/L)	4.2 ± 0.4	3.8 ± 0.4	4.0 ± 0.4	< 0.0001	0.784	< 0.0001
Total WBC count (10^9^/L)	6.367 ± 2.754	10.957 ± 4.301	7.632 ± 2.642	< 0.0001	< 0.0001	< 0.0001
Neutrophil count (10^9^/L)	4057 ± 2618	7762 ± 3841	4802 ± 2169	< 0.0001	0.0001	< 0.0001
Monocyte count (10^9^/L)	380 ± 156	632 ± 371	448 ± 202	< 0.0001	< 0.0001	< 0.0001
Lymphocyte count (10^9^/L)	1761 ± 716	2367 ± 1340	2118 ± 1004	< 0.0001	< 0.0001	0.028
Creatine kinase-MB (ng/mL)	-	7.1 (2.4 - 73.9)	2.4 (1.1 - 7.1)	-	-	< 0.0001
Troponin I (ng/mL)	-	1.1 (0.1 - 6.5)	0.1 (0.0 - 0.8)	-	-	< 0.0001
hs-CRP (mg/L)	0.2 (0.1 - 0.5)	3.7 (0.3 - 8.3)	2.2 (0.8 - 6.2)	< 0.0001	<0.0001	0.325

Data are expressed as mean ± SD, or median (interquartile range). STEMI, ST elevation myocardial infarction; HDL, high-density lipoprotein; LDL, low-density lipoprotein; BUN, blood urine nitrogen; eGFR, estimated glomerular filtration rate; WBC, white blood cell; hs-CRP, high sensitivity C-reactive protein.^ a^: Data were compared between ST elevation myocardial infarction patients and controls. ^b^: Data were compared between stable angina patients and controls. ^c^: Data were compared between ST elevation myocardial infarction patients and stable angina patients.

**Table 3:** Adjusted odds ratio and 95% confidence interval of stable angina and ST elevation myocardial infarction with TCF21 genotypic frequencies

Parameter	Controls (n=276)	Stable angina (n=381)	Crude OR	95% CI	AOR^a^	95% CI	p-value
***TCF21* genotypes**							
GG genotype^b^	56 (20.3)	58 (15.2)	1.00	-	1.00	-	-
GC genotype	153 (55.4)	184 (48.3)	1.16	0.76 - 1.78	1.61	0.87 - 3.02	0.133
CC genotype	67 (24.3)	139 (36.5)	2.00	1.26 - 3.21	2.49	1.24 - 5.07	0.010
GG/GC genotype^b^	209 (75.7)	242 (63.5)	1.00	-	1.00	-	-
CC genotype	67 (24.3)	139 (36.5)	1.79	1.27 - 2.54	1.76	1.04 - 3.02	0.036
	Controls (n = 276)	STEMI (n = 138)	Crude OR	95% CI	AOR^a^	95% CI	p-value
***TCF21* genotypes**							
GG genotype^b^	56 (20.3)	12 (8.7)	1.00	-	1.00	-	-
GC genotype	153 (55.4)	65 (47.1)	1.98	1.03 - 4.10	4.54	1.43 - 12.39	0.009
CC genotype	67 (24.3)	61 (44.2)	4.25	2.14 - 9.00	9.19	4.36 - 16.33	< 0.0001
GG/GC genotype^b^	209 (75.7)	77 (55.8)	1.00	-	1.00	-	-
CC genotype	67 (24.3)	61 (44.2)	2.47	1.60 - 3.82	4.44	2.05 - 10.09	0.0001

STEMI, ST elevation myocardial infarction; TCF21, transcription factor 21. AOR = adjusted odds ratio; CI = confidence interval. ^a^: The adjusted ORs with their 95% CI were estimated by employing multiple logistic regression models, after controlling for age, sex, smoking, body mass index, hypertension, hyperlipidemia, and diabetes mellitus. ^b^: as a reference group.

**Table 4:** Clinical and biochemical parameters of the studied patients with CAD in different genotypes of transcription factor 21

Parameters	GG	GC	CC	p-value
No.	70	249	200	
Age	64.0 ± 11.7	64.2 ± 11.4	64.8 ± 11.5	0.813
Sex (male/female)	54/16	196/53	161/39	0.810
Body mass index (kg/m^2^)	26.5 ± 3.9	26.1 ± 3.7	26.2 ± 3.7	0.726
Waist circumference (cm)	92.6 ± 8.3	91.9 ± 9.4	92.3 ± 10.9	0.862
Waist-to-hip ratio	0.94 ± 0.07	0.94 ± 0.07	0.94 ± 0.09	0.922
Systolic blood pressure (mmHg)	131 ± 21	133 ± 21	133 ± 21	0.874
Diastolic blood pressure (mmHg)	77± 13	78 ± 14	76 ± 14	0.351
Fasting sugar (mg/dL)	152.8 ± 92.1	143.9 ± 65.6	144.4 ± 77.3	0.678
HbA1C (%)	7.1 ± 2.1	6.8 ± 1.6	7.0 ± 1.7	0.472
Total-cholesterol (mg/dL)	178.5 ± 43.1	179.5 ± 43.7	175.0 ± 46.3	0.562
Triglyceride (mg/dL)	115.0 (85.5 - 178.3)	119.5 (89.0 - 179.8)	125.5 (93.3 - 190.8)	0.742
HDL-cholesterol (mg/dL)	39.3 ± 10.3	39.7 ± 11.0	40.2 ± 12.2	0.792
LDL-cholesterol (mg/dL)	106.6 ± 34.1	107.8 ± 39.6	103.0 ± 36.9	0.412
Uric acid (mg/dL)	6.2 ± 1.7	6.8 ± 2.4	6.6 ± 2.0	0.249
BUN (mg/dl)	17.6 (14.4 - 25.3)	17.8 (13.5 - 22.0)	17.7 (14.0 - 23.0)	0.082
Creatinine (mg/dl)	1.2 (1.0 - 1.4)	1.2 (1.1 - 1.4)	1.2 (1.1 - 1.4)	0.177
eGFR (ml/min/1.73m^2^)	66.9 ± 29.4	66.1 ± 24.6	62.8 ± 25.3	0.320
Hemoglobin (g/dl)	13.4 ± 2.2	13.8 ± 2.0	13.7 ± 2.2	0.528
Albumin (g/L)	3.9 ± 0.5	4.0 ± 0.4	3.9 ± 0.4	0.270
Total WBC count (10^9^/L)	8.341 ± 3.361	8.380 ± 3.520	8.747 ± 3.500	0.490
Neutrophil count (10^9^/L)	5218 ± 2797	5541 ± 3147	5709 ± 2857	0.500
Monocyte count (10^9^/L)	471 ± 216	492 ± 295	508 ± 249	0.592
Lymphocyte count (10^9^/L)	2235 ± 1155	2114 ± 976	2245 ± 1225	0.431
hs-CRP (mg/L)	1.9 (0.7-5.2)	3.1 (0.8 - 10.0)	3.2 (0.9 - 9.2)	0.032

Data are expressed as mean ± SD, or median (interquartile range). HDL, high-density lipoprotein; LDL, low-density lipoprotein; BUN, blood urine nitrogen; eGFR, estimated glomerular filtration rate; WBC, white blood cell; hs-CRP, high sensitivity C-reactive protein.
